# Open Source Tracking and Analysis of Adult *Drosophila* Locomotion in Buridan's Paradigm with and without Visual Targets

**DOI:** 10.1371/journal.pone.0042247

**Published:** 2012-08-09

**Authors:** Julien Colomb, Lutz Reiter, Jedrzej Blaszkiewicz, Jan Wessnitzer, Bjoern Brembs

**Affiliations:** 1 FB Biologie, Chemie, Pharmazie, Institut für Biologie-Neurobiologie, Freie Universität Berlin, Berlin, Germany; 2 Institute for Perception, Action and Behaviour, School of Informatics, University of Edinburgh, Edinburgh, United Kingdom; 3 Department of Genetics, Universität Leipzig, Leipzig, Germany; Imperial College London, United Kingdom

## Abstract

**Background:**

Insects have been among the most widely used model systems for studying the control of locomotion by nervous systems. In *Drosophila*, we implemented a simple test for locomotion: in Buridan's paradigm, flies walk back and forth between two inaccessible visual targets [1]. Until today, the lack of easily accessible tools for tracking the fly position and analyzing its trajectory has probably contributed to the slow acceptance of Buridan's paradigm.

**Methodology/Principal Findings:**

We present here a package of open source software designed to track a single animal walking in a homogenous environment (Buritrack) and to analyze its trajectory. The Centroid Trajectory Analysis (CeTrAn) software is coded in the open source statistics project R. It extracts eleven metrics and includes correlation analyses and a Principal Components Analysis (PCA). It was designed to be easily customized to personal requirements. In combination with inexpensive hardware, these tools can readily be used for teaching and research purposes. We demonstrate the capabilities of our package by measuring the locomotor behavior of adult *Drosophila melanogaster* (whose wings were clipped), either in the presence or in the absence of visual targets, and comparing the latter to different computer-generated data. The analysis of the trajectories confirms that flies are centrophobic and shows that inaccessible visual targets can alter the orientation of the flies without changing their overall patterns of activity.

**Conclusions/Significance:**

Using computer generated data, the analysis software was tested, and chance values for some metrics (as well as chance value for their correlation) were set. Our results prompt the hypothesis that fixation behavior is observed only if negative phototaxis can overcome the propensity of the flies to avoid the center of the platform. Together with our companion paper, we provide new tools to promote Open Science as well as the collection and analysis of digital behavioral data.

## Introduction

Controlling behavior is probably the most fundamental and ancestral function of nervous systems. A long tradition of entomologists studied how the insect thoracic ganglia, like the vertebrate spinal cord, can establish basic motor control [2], and how it is then further regulated by the brain [3]. The behavioral analysis of locomotion is greatly facilitated by automated or semi-automated methods for recording the position of an animal (or its body parts) over time. For example, our understanding of honey bee foraging and dance communication was boosted by the use of radar systems [4] and high-throughput software was developed to record locomotion in *Drosophila* [5]. Currently, there are a number of sophisticated free programs available that can track single or multiple individual walking flies from movie files. We can cite for instance Ftrack, (www.chronux.org), Ctrax (http://ctrax.sourceforge.net) or Flytrax, (http://code.astraw.com/projects/motmot). Ctrax and Flytrax provide full, open source access to their code. Together with our companion paper [6], we add to this arsenal of open source software tools for tracking larval and adult insect locomotion. Specifically, we provide here a straightforward method for online tracking of the centroid of a single adult fly without requiring the storage of any video information. Using the analysis software we also provide, our package is sufficient to describe a fly's locomotor activity in Buridan's paradigm [7], [1].

In recent years, the genetic toolbox for the *Drosophila* model system proved extremely useful in the search for the genetic and neuronal bases of behavioral control [8]. In pursuit of this research, different behavioral tests were developed to study fly locomotion [9]. One of the simplest of these tests is Buridan's paradigm [1], [7], where the flies walk between two inaccessible targets (stripes) in an otherwise homogeneously illuminated surrounding. By analyzing the walking speed of different mutant and transgenic flies, it was shown that the central complex [9]–[14] but not the mushroom body [15] neuropil regions need to be intact for the animal to have a normal walking speed. Some regions reveal their function in locomotor control during walking, while others only during development [16]. The behavior with regard to the stripes was quantified [17], [12], [13], and shown to depend on the peripheral retinula cells 1 to 6 [18]. Working memory was studied in a similar setup [19]. On the other hand, endogenous locomotion (without explicit stimuli) was studied in a circular arena [13], [20], [21] or in a square box [22].

Despite its apparent simplicity, Buridan's paradigm has failed to gain wider popularity. Among the obstacles encountered are the difficulties in setting up a tracking system and performing the necessary trajectory analysis. The most commonly used tracking software package for walking flies [5] (http://ctrax.sourceforge.net, http://code.astraw.com/projects/motmot) requires the acquisition and storage of the images at high resolution using one software and a further analysis of the video with another; and the metadata corresponding to each experiment need to be written independently. Moreover, many of the available tracking/analysis combinations require the commercial software Matlab (Mathworks, MA, USA). Here, we provide the community with an all in one, easily operable, open source tracking software that allows the experimenter to record the trajectory of one single animal in a circular arena, using inexpensive hardware without storing the video.

We build on mathematical tools developed in the free open source statistics package R (http://r-project.org) for field studies [23], [24], in order to analyze the trajectories of animals confined to a small platform. We also provide an easily operable interface, such that the analysis can be run with basic computer skills. In contrast to the tracking software, the analysis software is not devoted exclusively to Buridan's paradigm, but will be adapted to analyze any trajectory dataset.

In addition to this software package, we provide raw data and documentation files to ease the installation and encourage modifications of the software. In combination with inexpensive and readily available hardware (blueprints are provided along with the software online at http://buridan.sourceforge.net), this open source package enables the trajectories of walking flies to be gathered and analyzed. In order to demonstrate the potential of these tools, we compared *in vivo* (fly endogenous locomotion, or fly behavior in Buridan's paradigm) to *in silico* trajectories. Thus, together with the companion paper [6], we present a battery of new, open tools for improved animal behavior analysis.

## Materials and Methods

### Fly handling

Two- to five-days-old female flies of the Canton S strain (reared at 25°C, in a 12/12 hours light/dark regime at 60% relative humidity) had their wings clipped under CO_2_ anesthesia. They were then left undisturbed to recover overnight within individual containers, with access to water and sugar (local store), before being transferred to the experimental setup (modified from [7]) by gently tapping the opened individual containers. The experiment duration was set to 900 seconds. If the fly jumped into the water, tracking was automatically interrupted and the fly returned to the platform using a brush (see below).

### Experimental setup

**Figure 1:**
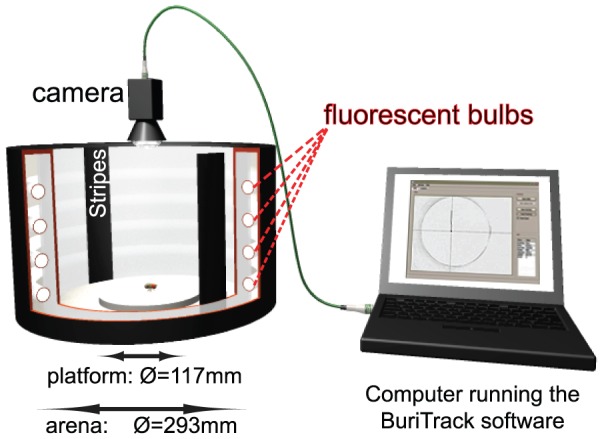
Inexpensive Hardware for Buridan's paradigm. The fly walks on a 117 mm platform surrounded by a water moat. The arena is homogenously illuminated, while stripes can be positioned on the inside of the arena wall. The fly is filmed from above and each frame is then treated by the tracking software.

The setup consists of a round platform of 117 mm in diameter, surrounded by a water-filled moat placed at the bottom of a uniformly illuminated white cylinder, 313 mm in height (Fig. 1). The setup was illuminated with four circular fluorescent tubes (Osram, L 40w, 640C circular cool white). Alternating current at >1 kHz was provided by an electronic control gear (Osram Quicktronic QT 1×40/230, discontinued, replacement product: QT-M 1×26–42). The four fluorescent tubes are located outside of a cylindrical diffuser positioned at a distance of 147.5 mm from the arena center. The temperature on the platform during the experiment was 27°C, and the luminosity ranged from 7.5 to 8 klx, with high intensity light from 370 to 850 nm. Except in the experiments where the flies were walking without explicit visual stimuli (‘endogenous locomotion’), stripes of black cardboard, either 30 or 50 mm wide, 313 mm high and 1 mm thick were taped on the inside of the diffuser. The retinal size of the stripes depended on the position of the fly on the platform and ranged from 57 to 74° in height (65° in the center of the platform). For narrow stripes, the width ranged from 8.4 to 19.6° (11.7° in the center of the platform); while the wide stripes were seen as 14 to 32° objects (20° in the center of the platform).

### Online downloadable tools

The free software package, its source code and the blueprints for the hardware setup are available for download at http://buridan.sourceforge.net. The enclosed documentation explains how to install the software, while a second file explains the structure of the analysis software and provides information to facilitate its modification and extension by users. Any modification can thus be easily performed with minimal computer skills.

### BuriTrack: a tracker for experiments in Buridan's paradigm

**Figure 2:**
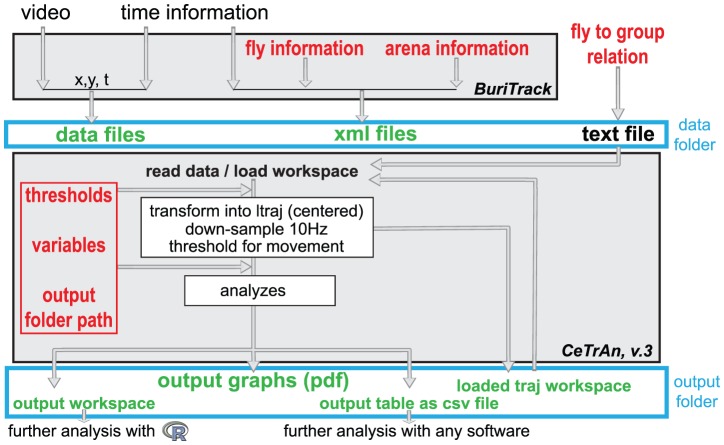
Software schematics. The experimenter enters information (in red) about the fly and the platform (semi-automatically) into the tracker application (BuriTrack). The tracker saves this information along with a time stamp in an XML file. Online analysis of the video leads to the extraction of the position of the fly over time, which is directly saved to the data file. The analysis software (CeTrAn) then reads a text file indicating the path to the XML file and the fly grouping information. It then automatically imports the data, transforms it into an easily workable class of data (*ltraj*) and performs the analyses following different variables the experimenter can set (in red). As outputs, CeTrAn writes R workspaces (before and after the analysis), a csv file of the computed parameters and pdf files where those metrics are plotted against the group factor.

The movement of flies was visualized via a standard commercial video camera (web cam). Any camera with a resolution of 640×480 or better will work (we used a Logitech Quickcam Pro 9000). The position of the fly is determined and recorded online (capture rate is determined by the speed of the computer). We obtained a mean resolution of one pixel for 0.35 mm (with a range of 0.31 to 0.4 mm). The user can observe the quality of the tracking via on-screen crosshairs placed on the image of the fly (Fig. 1). An alert is sounded and the recording stopped when the specified experiment duration is reached. The tracker was written in C++ using OpenCV (Willowgarage, http://opencv.willowgarage.com/) and Qt (Nokia, http://qt.nokia.com) libraries and operates as follows. Contrast and luminosity are set by the experimenter such that the fly appears as a dark spot on a homogenously bright background. Color information is discarded and a black-and-white image is generated using a user-adjustable threshold. The image is then inverted and a Gaussian blur is applied. The brightest dots are then determined (above a given threshold) and the brightest point in each dot is taken as the putative position of the fly. The parameters of the Gaussian and the threshold are adjusted in the interface (different parameters should be used for different spot sizes). If multiple points are located, the one closest to the position of the fly in the previous frame is taken (for the first frame, the brightest spot is taken). The coordinates of this point, which approximates to the centroid of the fly, is then saved along with the time stamp and the so-called burst number. The burst number is incremented and the tracking stops whenever the position of the fly is outside the platform limits or cannot be detected at all. In these instances, the recording is interrupted and an acoustic alert is played. Once the experimenter puts the fly back on the platform using a brush (or changes the camera settings) the recording can be resumed. The trajectory data is saved in an ASCII text file with four rows, separated by tabs: time (in ms), the two coordinates (X, Y in pixels), and the burst number (Fig. 2).

The user can modify thresholds and is asked to enter information about the experiment before it starts. In particular, the position of the platform is semi-automatically determined, by simply clicking on three points on the platform edge, spaced as far apart as possible. Any tilt of the camera can be visually detected and must be avoided during this step (the round platform has to fit the circle drawn by the software). This makes tilt correction [20] unnecessary. The information entered by the user (fly label, data file name, duration of experiment, stripes width and position, platform size and position, date and time at the start of the experiment and resolution of the camera capture) is saved in a separate text file (Fig. 2, encoded using the Extended Markup Language, XML). In order to sort individual experiments into experimental groups, the user lists the XML file name together with a group label in a separate text file.

### CeTrAn Centroid Trajectories Analysis software

CeTrAn, the analysis software, is written in the open source statistics package R (http://r-project.org) and can be used without computing knowledge thanks to a user-friendly interface written in RGG (R Graphical user interface Generator, http://rgg.r-forge.r-project.org). This interface allows the user to set different variables and to browse the disk to find the location of the three relevant entries: the folder containing the data, the group text file and an output folder into which the package will write its outputs (Fig. 2). In practice, data files are often dispatched in different folders; one then sets the “folder containing the data” to the parent folder, and adds the subfolder into the XML file name (for example: “experiment_1/fly_1.xml”). CeTrAn then imports and analyzes the data before drawing output graphs.

#### 1. Importing trajectories

Via the information specified in the group text file, CeTrAn reads the information contained in the data file and the corresponding XML metadata file. The information is then processed in the following steps: First, a table referring to each experiment is produced, tagging each fly with an identity (“id”) and a group tag. Then, the XML file corresponding to each experiment is read and its information is saved into the environment variable “env”. The corresponding trajectory is imported, transformed into elements of the *ltraj* class (one element for each burst) using the adehabitat package for R [23], and labeled with the fly identity. The coordinates are then transformed into an orthogonal coordinate system with its origin at the center of the platform and a unit of 1 mm, using the information contained in “env”.

The trajectories are down-sampled to achieve an evenly time-spaced trajectory of 10 data points per second (interpolated using a linear function). To reduce false positive movements due to camera noise or fly grooming, every movement smaller than 0.8 mm is discarded (i.e. larger than two pixels, see results section). A slightly modified version of the “mindistkeep” [23] function was used for this purpose. If the threshold distance is not reached, the data point is rewritten to be on the same spot as the previous one, and the distance to the next data point is recalculated. This threshold can be manually set, and was set to 0.8 mm if not noted otherwise.

#### 2. Extracting metrics

**Figure 3:**
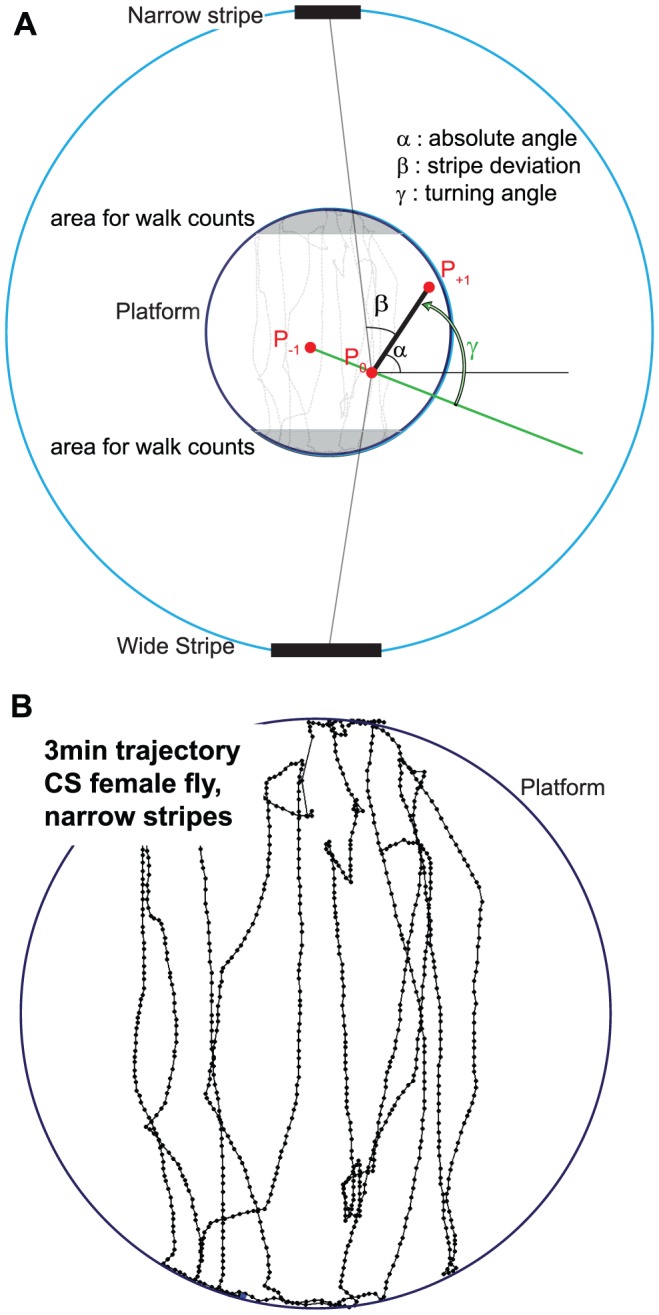
Calculation of angles and number of walks. **A**. The inner circle represents the platform, while the outer circle represents the arena and the light source (to scale). The bars represent the stripes (wide or narrow). Considering the movement from P_0_ to P_1_, α_0_ is the absolute movement angle (similarly α_−1_ is the absolute movement angle of the movement P_−1_ to P_0_). The turning angle γ can be calculated as α_0_ - α_−1_, it represents the change in direction at time 0. β is the “stripe deviation” angle, the angle from the movement to a vector going straight toward the middle of the stripe that is in the direction of the movement. In the “ltraj” object, α is assigned to P_0_, β to P_1_. Gray areas denote the sectors used to start and end a walk between stripes: a walk is counted for each passage from one gray area to the other. **B.** Trajectory example, zoomed on the platform size. The disposition of the stripes are at 90 and −90° as in A. Dots represent the position of the fly during the three first minutes of a test with narrow stripes, after down sampling to 10 Hz.

The *ltraj* class provides easy access to several manipulations of the trajectories and facilitates the process of data analysis. For instance, one has direct access to the distance between two consecutive data points, the angle of the velocity vector, as well as the turning angle, i.e. the angle between two consecutive velocity vectors (Fig. 3).

#### Median speed

Dividing the distance traveled by the time (always 0.1 s in our case, both given in the *ltraj* objects) gives the instant speed of each movement (in mm/s). We then report the median speed for each fly. Speeds exceeding 50 mm/s are considered to be jumps and are not included in the median speed calculation [21].

#### Walking distance

Adding up every movement length over the whole experiment yields the total distance traveled by each fly (in mm).

#### Turning angle

This parameter is directly read from the *ltraj* trajectories (Fig. 3). The median turning angle is calculated for each fly (in degrees).

#### Meander

The ‘meander’ is a measure of the tortuosity of the trajectories and is calculated by dividing the turning angle by the instantaneous speed [22]. Again, the median is calculated for each fly (in degrees×s/mm).

#### Centrophobism indices

We divided our circular arena into a smaller disk and an outer ring of equal surface (taking a disk of a radius √2 times smaller than the platform radius). The software then determines the proportion of time spent in each subdivision, treating data points while the animal is in motion independently from data points where the animal was stationary. The centrophobism indices for moving and for sitting (respectively) are then calculated as the difference between the number of data points outside and inside of the center area, divided by the sum of the two numbers. Therefore, an index of 1 means that the fly spent the entire experiment in the outer area, −1 is when the fly spent the entire experiment in the center and 0 denotes an equal distribution between outside and inside.

#### Stripe deviation

This metric corresponds to the angle between the velocity vector and a vector pointing from the fly position toward the center of the front stripe (Fig. 3). For each displacement, the vectors going from the fly position toward both stripes (situated at p(0,+/−146.5 mm) in the new coordinates centered in the platform center) are calculated and the respective angles between the velocity vector and each of those vectors are measured. Finally, the smaller of the two angles is chosen as output (corresponding to the angle with the stripe most in front of the animal, labeled ‘beta’ in Fig. 3, one stripe during one walk, and the other stripe during the next walk). The median of all deviation angles is reported for each fly (in degrees). Smaller values then correspond to a path directed toward the stripes.

#### Number of walks

This metric corresponds to the number of times the fly walked from one stripe to the other (closer than 80% of the platform radius toward the stripe, Fig. 3). The software detects when the fly enters one of the two areas and increments the count by one when it enters the opposite area. This process is reiterated until the trajectory ends.

#### Activity metrics

From the speed profile of the trajectory (instantaneous speed over experimental time), there are different ways to determine an activity pattern. Our first computation (time-threshold: indices labeled with (TT)) considers every movement as activity and every absence of movement lasting longer than 1 s as a pause (shorter periods of rest are considered as active periods). Changing this threshold from 1 to 0.5 or 1.5 s had little effect on the results (data not shown), such that we arbitrarily chose 1 s as standard.

In a second approach, we used the distance traveled by the fly in a sliding window of 1 second duration, measuring its mean velocity during that second (speed threshold: indices labeled with (ST)). When the speed was above the higher of two threshold (2.7 mm/s) the fly was classified as walking and when the speed was below the lower threshold (1 mm/s) the fly was classified as at rest. When the velocity was between the two thresholds, the fly maintained its previous classification until the second threshold was crossed [22]. In order to set these thresholds, we plotted a histogram of distance traveled in one second on a logarithmic scale (merging data from 60 wild type flies in the different stripe situations) and arbitrarily chose a value situated before the first and after the last minima of the histogram, respectively (Fig. S1). Setting threshold values to 1.15 and 2.5 mm did not alter the results (data not shown). The chosen values (1 and 2.7 mm) appeared to be very similar to the one chosen in another study, in order to fit the manually determined activity pattern (1 and 2.5 mm in one second) [21].

For both activity computations, we calculated for each fly the total activity time (in seconds), the number and median duration of the pauses and the median duration of bouts of activity (in seconds). For the median duration of activity (TT), we made a second calculation considering only activity bouts leading to larger displacement (>1 cm).

#### 3. PCA analysis

Built-in functions are used to perform a Principle Components Analysis (PCA; using the correlation matrix calculated with Pearson's method [25]). Correlation plots for each group of flies, indicating how the different metrics are correlated, are also generated. The activity metrics calculated with the speed threshold were discarded before performing the PCA (see result section). Although a PCA is automatically computed by the analysis package, the graphics presented in this paper were produced using a simpler graphical user interface (GUI) for R. We loaded the output workspace, restricted the number of groups or the number of variables taken into account, and produced 2D and 3D plots. Both scripts are available with the software in the “other_codes” folder. 3D objects were produced using rgl (http://rgl.neoscientists.org), from which snapshots or video files were generated.

#### 4. Outputs

The analysis software provides five different outputs. The first one is the workspace of the loaded trajectories, which can be reloaded into the analysis package. It allows the user to redo the analysis changing different variables without reloading the raw data, which is very useful to debug any novel analysis. The second output is the workspace resulting from the analysis. This can be loaded in R to perform further analysis, for instance, creating 3D plots of the PCA. The third output is a csv file including all metrics values for each fly. The table can then be imported into any statistical software. The fourth output is a pdf file in which the scores are plotted: Barplots representing means and standard errors for each group are given for each metrics. In addition, transition plots are drawn: the density of passage of the flies (every trajectory in a given group is taken into account) at each arena position (divided into 60×60 hexagons) is calculated and displayed via a color code. Its scale starts at 0 (blue) and increases to a value given by the 95%-quantile of the count-distribution (red). This scale makes small differences clearly visible and leaves only a few spots (5%), which are above the scale. One plot is generated for each group of flies tested, and a Gaussian blur of the data is added for a better visualization of the result (weights = 21, 16, 4, 1; the blur is done before the color scale is calculated). Finally, for the fifth output, a separate pdf file containing the analysis for each individual fly is generated. It gives the trajectory and a speed over time plot for each fly.

### Computer-generated data

We modified R code from the adehabitat package in order to generate trajectories staying within the bounds of the platform; we also added simulations of activity/inactivity patterns. We produced two types of data samples using different types of trajectory generation (‘correlated walk’ and ‘Lévy-walk’). In both cases the direction is set following a correlated walk rule (also in the Lévy-walk simulation): the first angle is chosen randomly, the next one is generated following a wrapped normal distribution around the previous angle. The correlation strength between two consecutive turning angles is determined by the variable “r”. The two types of computer-generated data differ in the way the walking speed is simulated: a step length for the 8999 movements (900 seconds at 10 Hz) was created by multiplying a Boolean variable simulating pauses (1 or 0, randomly generated using a uniform distribution with adjustable frequency “f”) with a speed value that was either created by drawing from a Chi distribution around a mean value “h” (correlated walk) or by drawing from a Lévy distribution:


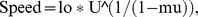


where U is a uniform distribution between 0 and 1, mu was set to 2.6.

The position is then calculated iteratively, starting at the center of the arena. When the position lands out of bounds, it is replaced by the nearest point within the arena limits and the angle sequence is recalculated taking the first angle randomly. Accordingly, the next movement will lie outside of the platform with a probability >0.5.

We then fitted the variables in order to approach the fly endogenous locomotion data. We set the frequency “f”, the angle correlation “r” and the speed variables “h” or “lo” in order to fit the activity time (TT, about 33% of the time active), the turning angle (8.3°) and the median speed (13.8+/−0.4 mm/s). Since these variables interact, we had to adjust them iteratively. We finally set for our correlated walk: f = 15%, r = 0.9965, h = 0.7±0.3; and for our Lévy-walk: f = 12%, r = 0.9963, lo = 0.8±0.4. The frequency is low because any short pauses are considered as active periods. The angle correlation is high because turning angles are calculated only between two velocity vectors, while the angle correlation calculates an angle difference also when the speed is zero (85% of the time for correlated walk). The speed variables were set to vary in order to fit the median speed variability and to get some variation between the 20 artificial walks, which is necessary for a correlation analysis (13.9+/−0.6 mm/s for Lévy-walk, 13.4+/−0.4 mm/s for correlated walk).

### Statistics

Calculated metrics for individual flies were either single values (for instance for the total distance traveled) or the median of multiple values (for example for the median of all instant speed values). We chose to use medians instead of means to describe these latter locomotion characteristics because their histograms clearly showed non-normal distributions. The analysis package then plots the means and standard errors of these medians, since their distribution across flies appeared to approximate a Gaussian distribution. We performed a MANOVA test on the entire dataset, in order to look for significant differences.

## Results

### Video tracking accuracy

We used readily available hardware (Fig. 1) and a custom-coded open source free software package (Fig. 2) in order to study the locomotion of fruit flies which were rendered flightless by clipping their wings. As for the larval tracker system presented in the companion paper [6], one individual animal is tracked while moving in an open field environment. Since we did not eliminate the background image (no background subtraction is performed), the tracker works only with a uniform white background in its present form and special care has to be taken in order to optimize the contrast and luminosity of the input image. In addition, the level of water in the moat must be adjusted to prevent the platform edge from appearing as dark spots. Online comparison of the fly movement and the recorded coordinates, which are both visible in the image provided by the tracking software (see Fig. 1), allowed us to visually confirm that the quality of the recording was sufficient for our experiments. Analysis of the trajectories also showed that the spatial resolution needed to be further restricted to avoid false positive movement (see below).

Since the fly centroid may move without the fly actually walking (due for example to camera noise or fly grooming), the tracker records small false positive movements. Low-pass filtering of the data is not sufficient to get rid of these artifacts, as can be observed by looking at the distribution of “speed angles” (α in Fig. 3), where we noticed bumps at multiples of 22.5° (data not shown, probably due to 2 pixels wide displacement of the recorded position). We eliminated these artifacts *offline* in CeTrAn, by setting a threshold of 0.8 mm (one pixel is less than 0.4 mm wide, a fly is about 2 mm long) under which a movement is discarded. The “total distance traveled” metric is not affected by this procedure, while the “median speed” is (see Material and Methods).

### Similar activity metrics yielded by time and speed thresholds computations

**Figure 4:**
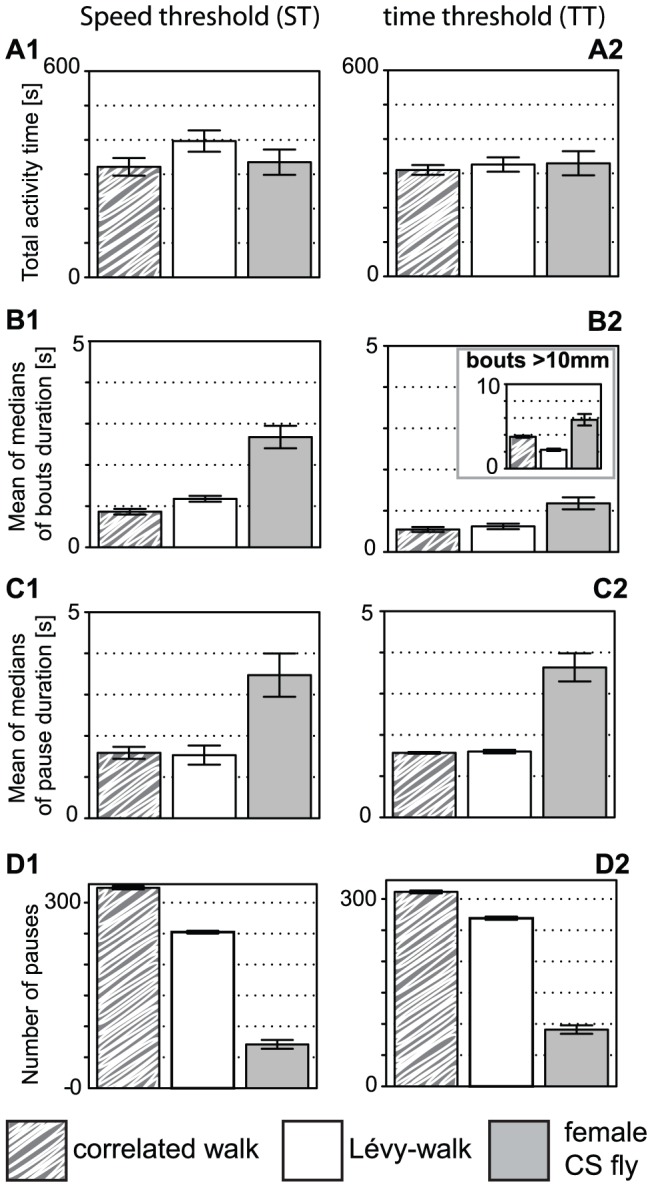
Activity calculations using different computations give similar results for endogenous locomotion and computer-generated data (grouping codes given below the graph). Pause-activity patterns were determined using either a speed- (left, labeled **1**) or a time threshold (right, labeled **2**). **A.** Total activity time represents the time the animal is considered active. **A2** was set to be similar in the computer-generated data. **B.** Duration of activity periods. Inset represents the same calculation as in B2 but considers only activity bouts leading to a displacement of 1 cm or more. **C.** Duration of the pause periods. **D.** Number of pauses. Bars represent means and error bars standard errors, n = 20 in each group.

Different algorithms can be used to decipher the activity pattern of a fly. We used one independent of the fly speed and one used in previous studies. We used either a time threshold for pauses (absence of movement for more than one second is considered as a pause, every movement starts an activity period) or two speed thresholds for activity (displacement length during one second determines whether the middle data point is considered as active following a two thresholds rule, see Materials and Methods [22], [21]). The two activity calculations lead to clearly comparable metrics (Fig. 4): differences between groups appear independent of the calculations used (Fig. 4 and data not shown). In contrast, the correlation analysis (see below) revealed differences in the two computations. This validates the use of our speed-independent calculation. Furthermore, the discovery of mutant flies showing differences in activity patterns or in which the two metrics would differ, may help to design better algorithms.

### Computer-generated data as random walks

The most effective way to test the analysis software is to feed it with data of known characteristics. We therefore modified R adehabitat functions [23], in order to generate both ‘Lévy-walks’ and ‘correlated walks’ restricted to the platform area (see Materials and Methods). These trajectories proved to be essential for determining the chance level for the stripe deviation metric and the centrophobism indices, the theoretical chance values of which are difficult to calculate (the stripe deviation angles can vary from 0 to 120° depending of the position of the fly, and only the use of computer generated data allowed us to set the chance value for their median to 45°). In addition, we used these trajectories to give an estimate of the mathematical correlation of the different metrics. For the latter purpose, we fitted the computer-generated data such that the total activity time (TT), the median turning angle and the median speed would approach values obtained from flies' endogenous locomotion (while the threshold for movement was applied to all data types). We also introduced variability in the speed, such that the error bars would also fit to fly error bars. This allows the artificial data to be in the same range of values as the fly data, allowing easier comparisons.

**Figure 5:**
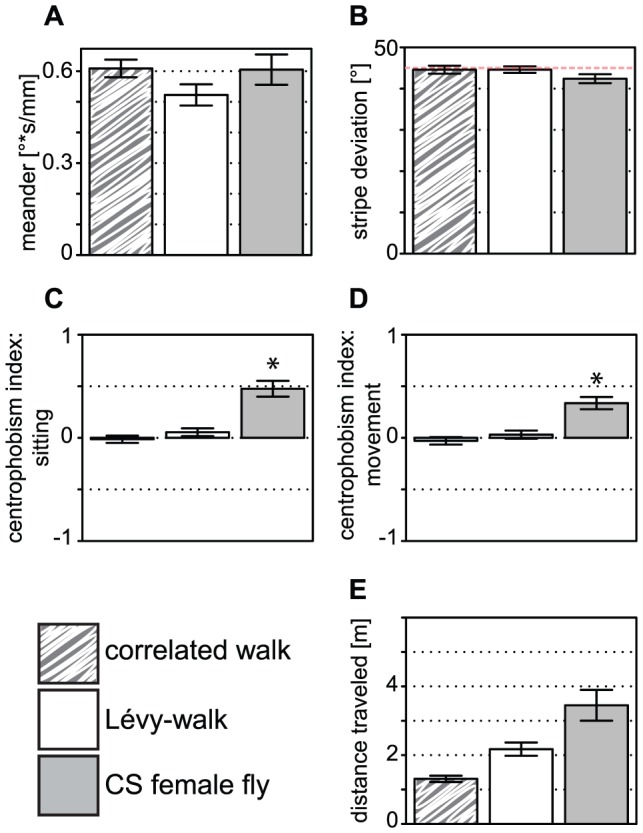
Trajectory parameters of real flies and computer-generated data (grouping codes given below the graph). Both **A.** Meander (turning angle divided by speed) and **B.** stripe deviation are similar in fly and computer-generated data. Red line denotes 45°, the mean value for computer-generated data. **C–D.** Centrophobism score for sitting (C) or for moving (D) is positive only for fly data. **E.** The distance traveled is different between the three types of data. Bars represent means and error bars standard errors, asterisks denote significant differences after a MANOVA analysis, n = 20 in each group.

The use of two types of computer-generated data allowed us to point to interesting features of the algorithms. Correlated and Lévy-walks, albeit made to differ only in their speed pattern, surprisingly show differences in other unrelated metrics: Lévy-walks show a longer activity bout duration (Fig. 4B) and a longer distance traveled than correlated walks (Fig. 5E). These results are due (see Fig. S2) to the higher amount of small discarded movements in correlated walks (that increase the number of small pauses) and to the fact that Lévy-walks show more instances of high speeds classified as jumps (these are not included in the median speed calculation, but included in the total distance traveled; importantly, our fly dataset does not contain any of these jumps). The total distance traveled is still much shorter than that of fly data (Fig. 5 E). The computer-generated data indeed contains numerous short pauses not reaching the 1 s threshold value. The correlation analysis is also affected by the discarding of small movements, because the speed variable then affects the number of non-moving points. Since Lévy-walks show so many jumps, we did not use its correlation matrices. In addition, the main source of variation in the computer-generated data is the speed variable (see Materials and Methods), such that some weak correlations are boosted. Therefore, the correlation matrix for computer-generated data had to be analyzed with caution (see below).

### Endogenous locomotion versus computer-generated data

Three metrics of the computer-generated data were fitted to the fly endogenous locomotion data (trajectories in the absence of visual targets), and three additional variables were similar between the computer and the fly data (i.e. no significant differences between the three groups in a MANOVA): total activity time (ST, Fig. 4 A1), meander (Fig. 5 A) and stripe deviation (Fig. 5B). None of them was unexpected, since the meander is calculated from two fitted parameters, the total activity time (ST) is highly dependent on the fitted activity time (TT) and the stripe deviation should be at chance level in all three groups. In contrast, the activity patterns of the flies appear non-random: the duration of the activity and pause periods is much larger than for the computer-generated data (Fig. 4 B, C).

Besson and Martin have shown that flies trapped in a box walk along the walls [26]. They interpreted this behavior as both thigmotaxis (hugging the wall due to mechanosensory stimuli) and centrophobism (avoidance of the center due to visual inputs) [26]. There is no wall in our setup, but flies nevertheless have the propensity to walk at the platform edge, as previously reported [20]; we thus called our quantification “centrophobism index”, although we cannot exclude touch as a relevant sensory input. We independently calculate a centrophobism index for moving (selecting position with positive displacement) and an index for sitting (taking data points where the fly did not move). One cannot *a priori* exclude that an increased probability of finding data points at the periphery is not due to chance. Therefore, analyzing the computer-generated data is instructive. Reaching the platform border, the next computer-generated data point has indeed more than a 50% chance to lie outside of the platform for the next data point (see Materials and Methods). The trajectory may thus be stuck at the platform border. However, this effect (higher probability to stay at the border when reached) turns out to be negligible and barely compensates for the initial start in the center of the platform (Fig. 5 C, D). Chance levels for the centrophobism indices appear to be zero and the positive indices of the fly data have thus a biological cause.

**Figure 6:**
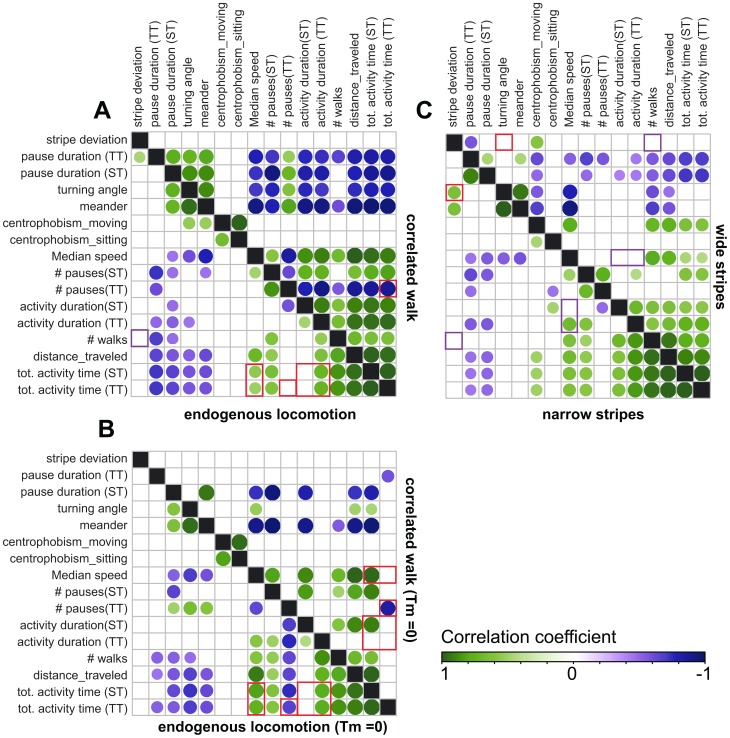
Correlation plot for the different groups of data. The order of the variables was set by clustering them for endogenous locomotion data (A, lower left part). Only significant correlations are shown. Each matrix is divided into two halves representing the correlation in different groups as indicated below and on their side. Movements smaller than 0.8 mm were discarded in A and C, but not in B, Tm -“Thresholds for movement”. Positive correlations are represented by green dots, negative correlations by violet dots. The size and color of the dots represent the correlation coefficient, as indicated. **A.** Correlation matrices of endogenous locomotion (lower left half-matrix) and computer generated correlated walk (upper right half-matrix). **B.** Same as in A without discarding movements smaller than 0.8 mm **C.** Correlation matrices of fly data in Buridan's paradigm, with narrow (lower left half) and wide (upper right half) stripes. Highlights in A and B: **Small red squares**: The number of pauses is not correlated with the total activity time in the real fly data. **Elongated red rectangles**: The median speed is correlated with the total activity time (TT) in the fly data. **Large red squares**: The duration of activity bouts (TT but not ST) correlates with the total activity time (ST and TT) in the fly data. Highlights in A and C: **Small purple squares**: The number of walks is not correlated with stripe deviation. **Small red squares in C**: The angle deviation and stripe deviation are correlated only in the narrow stripes situation. **Elongated purple rectangle** in C: The median speed correlates with the duration of activity periods in the narrow stripes situation. n = 20 flies for each group.

**Figure 7:**
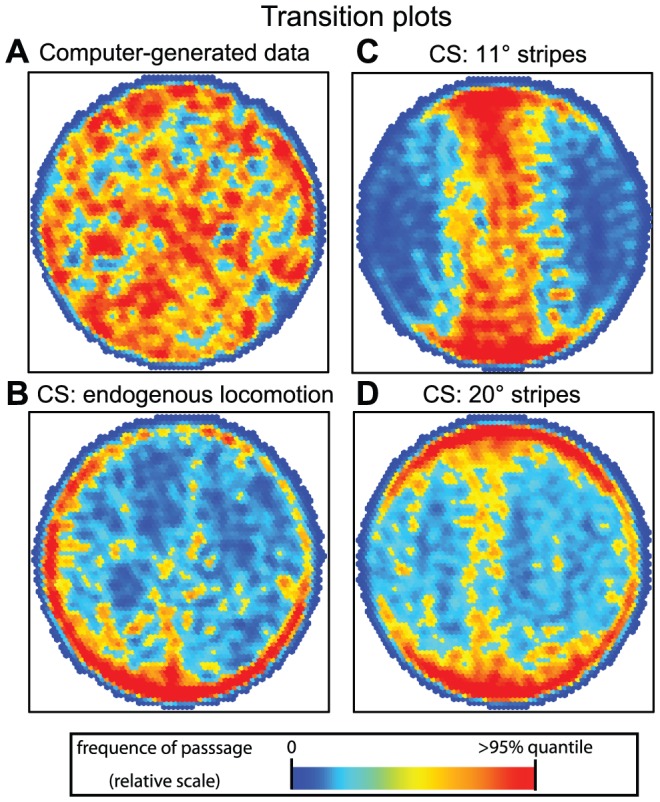
Transition plots for the different groups of data. The relative frequency of the fly passage at each position is plotted (red denotes a frequency above the 95% quantile value, dark blue means flies were rarely present. White indicates that none of the flies ever transitioned through this position). **A.** Computer-generated data (here correlated walk, but Lévy-walk transition plot is nearly identical) **B.** Endogenous locomotion. **C.** Buridan's paradigm with narrow stripes (11°). **D.** Buridan's paradigm with wide stripes (20°). n = 20 in each group.

We then uncovered the dependence of the different metrics on each other. In order to differentiate between mathematical and biological relationships between metrics, we compared the correlations among them in the fly endogenous locomotion and in the computer-generated correlated walks. To this end, we generated correlation matrices of the trajectories either discarding (Fig. 6A) or including (Fig. 6B) particularly small movements. In the fly data analysis, the inclusion of these movements leads to a positive correlation between the number of pauses and the turning angle (Fig. 6B), which indicates that most of these movements are indeed false positives. On the one hand, these movements split pauses by introducing short activity periods, thereby increasing the number of pauses, while on the other hand, their direction being random, they increase the median turning angle. Interestingly, the activity parameters computed via the speed threshold, but not the ones computed via the time threshold, show correlation with the median speed. In contrast, correlations between the number of pauses and other activity metrics differ for the two calculations, indicating that they are not equivalent. Using this cautious approach, two interesting conclusions can be drawn from the observations that some variables, independently generated in the artificial data, show correlation in the fly trajectories (Fig. 6A, B): First, the total activity time (TT) is correlated with the duration but not the number of activity periods (identical to the number of pauses). Second, the median speed is correlated with the total activity time (TT), suggesting that these two features (how often and how fast to walk) are not independently controlled by the fly nervous system,

In Fig. 7, transition plots are shown. They represent the frequency of passage of flies at any location on the platform. The centrophobic behavior of flies in endogenous locomotion can be clearly seen, while they seem to move preferentially on one side of the arena (Fig. 7B), suggesting that our environment was not perfectly homogenous. It still needs to be tested whether this is due to chance, to imperfections in the setup or to stimuli from the experimental room.

### Effect of visual targets on fly behavior

**Figure 8:**
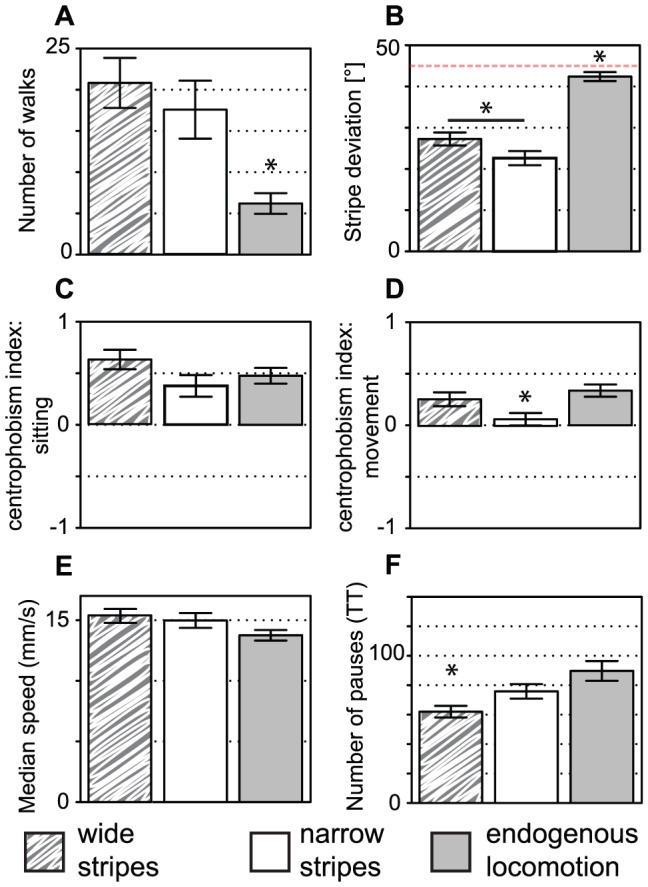
Fly trajectory metrics for endogenous locomotion (gray bars), Buridan's paradigm with narrow stripes (white bars) or wide stripes (striped pattern). **A.** In the presence of visual targets, the fly shows more walks between the stripes than in their absence. **B.** Median stripe deviation is different in the three groups. Red line denotes the value for random walks **C.** Centrophobism during pauses is still present in all three groups. **D.** Centrophobism while moving is eliminated by narrow stripes. **E.** Median speed is not significantly affected by visual targets. **F.** The number of pauses is lower in the wide stripe condition as compared to the two other conditions. Asterisks denote significant differences after a MANOVA analysis. Bars represent means and error bars standard errors, n = 20 in each group.

We also tested flies in Buridan's paradigm, where narrow or wide (11° and 20°, respectively) stripes form black targets on opposite sides of the illuminated arena. In similar situations, flies were reported to perform direct walks back and forth between the two stripes, a behavior which was explained by alternating fixation and antifixation of the stripes [1]. From the transition plots, this stereotypic behavior is apparent only when narrow stripes are present (Fig. 7), although both target types induce similar numbers of walks (Fig. 8A). The behavior toward the stripes is best described with the median stripe deviation metric, which is significantly different in the two situations (Fig. 8B, MANOVA: F = 4.3, p<0.05). Interestingly, the number of walks is not correlated with the deviation from the stripe, but with the total distance traveled (Fig. 6A, C). This difference between the effect of narrow and wide stripes is reflected in the difference in the correlation coefficient between stripe deviation and turning angle, which is high only in the narrow stripes situation (Fig. 6C).

The centrophobism indices also reflect this response to the stripes, albeit indirectly. While flies preferentially stay at the outer half of the platform during their pauses in all three situations (Fig. 8C), the centrophobism index for moving is reduced to almost zero by the presence of narrow stripes but hardly affected by wide stripes when compared to endogenous walk (Fig. 8D, MANOVA, F = 0.8, p>0.3). However, this latter metric is then correlated with the stripe deviation and the median speed (Fig. 6C) while these correlations are absent in endogenous locomotion data (Fig. 6A). This indicates that flies that are particularly responsive to the wide stripes also enter the center area of the platform more often.

We expected flies going straight towards the stripes to walk faster. However, this correlation can be found only when wide stripes are used (Fig. 6A, C), and the median speed appears largely unaffected by the presence of the stripes (Fig. 8E, MANOVA, F = 2.5, p>0.08). In addition, activity parameters are only marginally affected (Fig. S3), with the number of pauses being lower in the wide stripe situation (Fig. 8F, MANOVA; F = 7.4, p<0.01). However, the fixation/antifixation behavior seen with the narrow stripes seems to induce a coupling of speed and duration of activity bouts (Fig. 6C). This may indicate that longer activity bouts with faster walking speed are more frequent when the fly is walking between stripes.

### Principle components analysis

A PCA (principal component analysis) represents the data along its most variable axes. This procedure allows the dimensionality of the data to be reduced with a minimal loss in information content. It is important to keep in mind that it does not optimize differences between groups, but allows the user to visually compare the different groups based on the totality of the metrics computed. In order to avoid an overestimation of highly correlated metrics in the PCA (for example, using the same variable multiple times would overestimate its impact on the total data variance), we had to discard one type of activity metrics. Since the calculation using the speed thresholds are mathematically correlated with the fly speed (see above), we chose to discard these parameters before performing the PCA.

**Figure 9:**
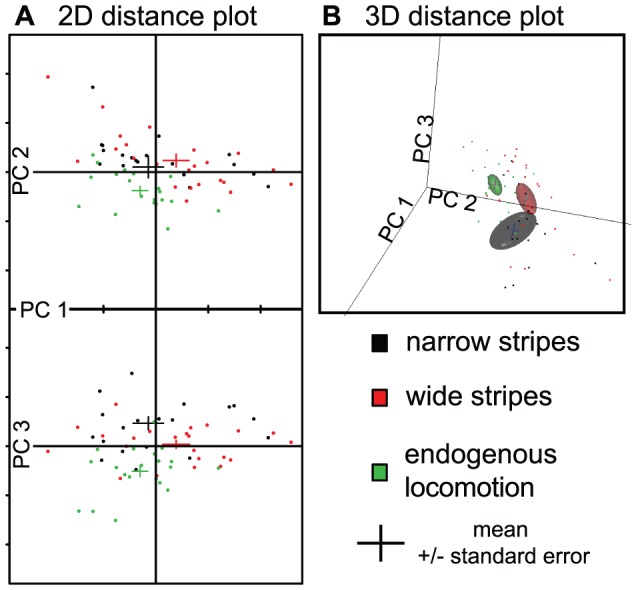
Reduced state space of fly locomotion behavior after PCA separates the three experimental conditions. **A.** 2D scatter plot showing each fly as a dot, and the mean and standard error of the factor loadings as bars. Abscissa is the first principal component (PC1). Ordinate is PC2 on the upper panel and PC3 on the lower panel. **B.** Snapshot of the corresponding 3D representation plot. Each dot corresponds to a fly coordinate, bars are means and standard errors, and the ellipsoids represent the 80% confidence interval (calculated from the covariance using the loadings on the three first principal components).

Performing a PCA on the fly trajectories in the three situations (Fig. 9, Movie S1), the first principal component shows the highest loadings for the activity variables (duration of activity bouts, activity time, median speed, distance traveled and number of walks), while the other variables contribute differently to the second and third principal components. All three principal components together explain 71% of the variability (41, 18, and 13%, respectively). Interestingly, the different groups differ only slightly in PC1 (Fig. 9), confirming that the activity is only marginally affected by the presence of the stripes. In contrast to what one would conclude looking only at the transition plots, the two groups with stripes are closer together in the reduced state space of the PCA, while the endogenous locomotion group stands alone.

## Discussion

Using a sample of 20 female Canton S fly trajectories in each of three different conditions (without visual targets, with narrow stripes and with wide stripes), together with computer-generated data on our new analyzing system, we were able to reproduce published results as well as discover unexpected characteristics of fly locomotion. Our tracker is able to follow the trajectory of a fly with an adequate spatial (0.4 mm/pixel) and temporal (about 20 Hz) resolutions. Both resolutions were indeed restricted before the analysis (0.8 mm and 10 Hz). By analyzing trajectories of flies in the absence of visual targets, we could conclusively show that the previously described centrophobism behavior [26] can also be observed in flies with clipped wings and in the absence of walls. Importantly, the analysis of artificial trajectories demonstrates that positive scores cannot be explained by randomly generated stops at the platform border. As expected from previous studies [27], [28], the activity pattern of the fly cannot be simulated via a simple uniform proportion of pauses. A rough plotting of pause duration frequency may indeed suggests that pause duration has a fractal structure (data not shown), as previously stated [22], [28]. Finally, our correlation study indicates that the most active animals are also the fastest, suggesting that the two trajectory properties may be subjected to similar biological constraints.

In our setup, the flies modified their behavioral reaction to the visual targets. This can be clearly seen in transition plots (Fig. 7) and, even better, in the PCA representation (Fig. 9), which will likely be a convenient means of comparing different fly strains. On the other hand, plotting each metric independently allows for more precise interpretations. Indeed, the transition plots suggest that narrow but not wide stripes induce a strong fixation/antifixation behavior (going straight toward and then away from one stripe, toward the other stripe), as expected from previous reports [1], [29]. This is mirrored by the centrophobism index for moving, which is reduced to zero only in the narrow stripe situation. Interestingly, the centrophobism index for sitting is unaffected, suggesting that this latter score can measure the centrophobic behavior in flies engaged in Buridan's paradigm. The correlation analysis is another informative tool: for instance, only strong fixation behavior seems to lead to a correlation between the median turning angle and the median stripe deviation.

The effect of wide stripes on fly behavior was unexpected. It appeared that it cannot fully overcome the centrophobism effect. Previous studies simulating object targeting [30] postulated that flies aim at the stripes' edges. Indeed, the analysis of trajectories of flies from a different species approaching a stripe showed that they aimed for the stripe's edge [31]. However, using a very similar approach, we could not see any preference for the stripes' edges over the stripes' center in our raw trajectory data. Although our setup gives no direct information about the gaze of the fly, the latter observation suggests that fruit flies may not target a specific sector of the wide stripes. Consequently, in the wide stripe situation, stripe fixation is severely degraded. Maybe, as was previously hypothesized in another study [12], fixation behavior arises from negative phototaxis, since wingless flies do lose their positive phototaxis [32] (and unpublished results). Indeed, experiments using wider stripes of different height and manual recording of one single movement from the center of the platform to its edge suggested that flies show both a preference for dark against white areas, and a preference for the edges [29]. Interestingly, the presence of edge preference depends both on the stripe width and height. For 60°high stripes, edge preference seems to appear only with 70° wide stripes. One may postulate that an interaction between negative phototaxis and centrophobism might translate into strong fixation behavior only when stripes are narrow, maybe because the larger bright area (the area not covered by a stripe) is a stronger aversive stimulus, overcoming centrophobism.

Freely moving animals have been shown to follow different locomotion strategies depending on their goal. For instance, the roundworm *C.elegans* changes its behavior from a correlated walk towards a Lévy-walk after some time spent without finding food (area-restricted search, [33]). In a first approach to analyze the fly walking patterns, we produced artificial correlated walks and Lévy-walks and compared them to each other and to fly trajectories. The analysis revealed little difference between the two computer-generated walks, while the differences to fly data were informative with regard to the centrophobism of flies and their activity patterns (see above). The algorithms we adapted from the literature [23] for generating the walks vary the speed according to different distributions, leaving the turning angle calculation unaffected (a wrapped normal distribution). In order to simulate Lévy-walks more accurately, one may have to work on the generation of the turning angle, keeping the speed more constant. Despite this caveat, the simulated data series proved useful. They are excellent tools to debug evaluation algorithms and to set chance values for the metrics, as well as for the metrics correlations: they allowed us to exclude a mathematical cause for the correlation between walking parameters.

Using a random walk algorithm (similar to the correlated walk we used here if one set the correlation variable to zero), Götz and Biesinger could reproduce the centrophobism effect [34]. The key difference in their algorithm is its behavior while reaching the platform border: while ours stops and produces a random angle, theirs continues to walk at the border for the distance set by the velocity vector. As stated by the authors, such simulations are not very informative about the actual strategy used by flies; only a careful study of the fly trajectory will yield better insight.

In the last decade, different tracking and analysis solutions have been developed in order to test flies specifically in Buridan's paradigm, but unfortunately they were never made public using a modern repository. Our package, consisting of BuriTrack, the trajectory recorder, and CeTrAn, the analysis software, is open source and cross-platform, i.e., it can be used under Linux, OSX, or Microsoft Windows, and necessitates only inexpensive hardware and no programming skills to be run. So far, BuriTrack works only in a homogenously white environment (it does not implement background subtraction), while CeTrAn was designed to be used with any 2D trajectory data with little modification. In order to facilitate such data sharing, we are working on the importing and exporting functions of CeTrAn and on the development of an open trajectory database using data sharing platforms (http://figshare.com), where additional reference data will be published.

Since R is widely used in the bioinformatics community, we are confident we will see improvements to CeTrAn in the future. First, we ourselves will implement additional algorithms previously used in the analysis of Buridan's paradigm experiments but not fitted for the analysis of endogenous locomotion [9]. Second, with the package, we provide a growing database of computer-generated and fly walk examples (already available on http://buridan.sourceforge.net), in order to facilitate the testing of new algorithms even without access to hardware and/or flies. For instance, mathematicians may study the putative fractal structure of pause durations, which is beyond the mathematical competence of most biologists [27].

In conclusion, we present here an open source free software package that can easily be implemented in any laboratory for teaching or research purposes. The paradigms described here can uncover features of animal locomotion and its modulation by external stimuli, like the dependence of the fixation/antifixation behavior of flies in Buridan's paradigm on stripe width. Together with the larval trajectory tracker described in the accompanying paper [6], we hope to further popularize the analysis of *Drosophila* locomotion.

## Supporting Information

Movie S1 **Movie of the 3D representation shown in** **Fig. 9B** **, see** **Fig. 9** **legend for more information.**
(AVI)

Figure S1 **Histogram of speed frequency (on a logarithmic scale, speed is calculated on a sliding window of 1 s) for all 60 flies tested.** Bars denote the lower and upper thresholds (1 and 2.7 mm/s, in red and green, respectively).
(EPS)

Figure S2 **Distribution of speed values frequency in the computer-generated data, when no threshold for movement (Tm) is applied.** Red bars represent the threshold for movement (8 mm/s = 0.8 mm in 0.1 s) and for jumps (50 mm/s) that delimit the grayed areas of discarded movements.
(EPS)

Figure S3 **Activity in fly endogenous locomotion (gray bars), in Buridan's paradigm with narrow stripes (white bars) or wide stripes (striped pattern).** **A**. The number of pauses is significantly different between the wide stripes situation and the two other situations. **B–D**. No significant differences in the total activity time (B), the median activity bout duration (C) and the median pause duration (D) in the three situations, although a trend for higher activity can be seen in the wide stripe situation. Bars represent means and error bars standard errors, n = 20 in each group.
(EPS)
