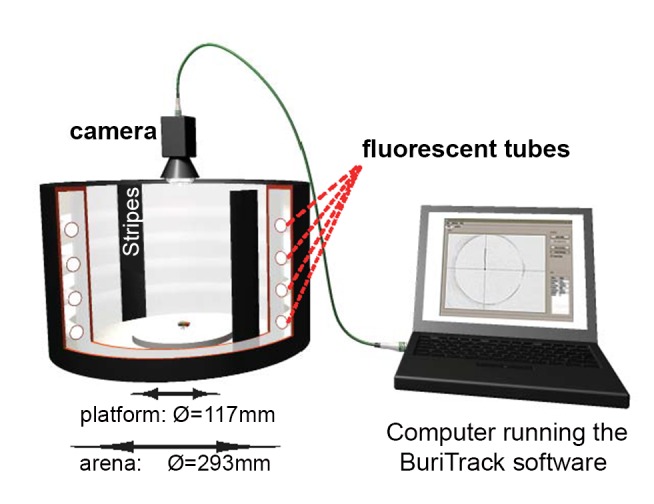# Correction: Open Source Tracking and Analysis of Adult *Drosophila* Locomotion in Buridan's Paradigm with and without Visual Targets

**DOI:** 10.1371/annotation/41b2d3fd-e816-420c-80d0-88290796b1cd

**Published:** 2012-11-06

**Authors:** Julien Colomb, Lutz Reiter, Jedrzej Blaszkiewicz, Jan Wessnitzer, Björn Brembs

There were numerous errors throughout the article.

The fifth author's name is misspelled. The correct name is: Björn Brembs.

Reference 6 is incorrect. The correct reference is: Gomez-Marin A, Partoune N, Stephens GJ, Louis M (2012) Automated Tracking of Animal Posture and Movement during Exploration and Sensory Orientation Behaviors. PLoS ONE 7(8): e41642. doi:10.1371/journal.pone.0041642 http://www.plosone.org/article/info%3Adoi%2F10.1371%2Fjournal.pone.0041642

Figure 1 is incorrect. The correct figure can be viewed here: 

**Figure:**